# Prevalence of Latent Tuberculosis Infection in the Middle East and North Africa: A Systematic Review

**DOI:** 10.1155/2021/6680651

**Published:** 2021-01-28

**Authors:** Mazin Barry

**Affiliations:** Department of Internal Medicine, Division of Infectious Diseases, College of Medicine, King Saud University, Riyadh, Saudi Arabia

## Abstract

**Objective:**

Data on the prevalence of latent tuberculosis infection (LTBI) in Middle Eastern and North African countries are scarce. We aimed to review all relevant published data in countries belonging to this region to determine the overall prevalence of LTBI in the Middle East and North Africa (MENA) region.

**Methods:**

In this systematic review PubMed and Google Scholar databases were searched for observational, prospective, retrospective, cross-sectional, and cohort studies providing prevalence data of LTBI in any MENA country. Studies fulfilling the search criteria were incorporated in the review. Overall prevalence of LTBI with 95% confidence intervals (CI) was calculated using the random-effects model; heterogeneity was assessed using *I*^2^ statistics. Gender and age group-based subgroup analyses were performed to evaluate the basis of heterogeneity.

**Results:**

The total number of overall LTBI studies identified was 956, of which 31 studies from ten countries within the MENA region were included that represented 12,439 subjects. The overall prevalence was 41.78% (95% CI 31.18% to 52.78%, *I*^2^ = 99.31%). By gender-based subgroup analysis, the prevalence of LTBI was 33.12% (95% CI 18.97% to 49.04%, *I*^2^ = 99.25%) and 32.65% (95% CI 19.79% to 47%, *I*^2^ = 98.89%) in males and females, respectively, while in the age-based subgroup analysis, the prevalence of LTBI was 0.44% (95% CI -0.05% to 0.9%), 3.37% (95% CI 2.23% to 4.74%, *I*^2^ = 0%), and 43.81% (95% CI 33.09% to 54.82%, *I*^2^ = 99.18%) for children, adolescents, and adults, respectively.

**Conclusion:**

This systematic review reveals a high prevalence of LTBI in the MENA region; enhanced LTBI surveillance and prompt infection prevention steps are urgently needed to prevent active tuberculosis, this would help achieve the World Health Organization End TB Strategy 2035, and the United Nations Sustainable Development Goals 2030 target in the MENA region.

## 1. Introduction

Tuberculosis (TB) is a major health problem, with an estimated 10 million people (range 9 to 11.1 million) developing TB disease in 2018, of which 5.8 million, 3.2 million, and 1 million were men, women, and children, respectively. Two-thirds of cases were from eight countries, India (27%), China (9%), Indonesia (8%), Philippines (6%), Pakistan (5%), Nigeria (4%), Bangladesh (4%), and South Africa (3%) [1]. Latent tuberculosis infection (LTBI) does not induce infectious expression of the disease, although it causes continuous immune response generated towards TB antigens. LTBI has a 10% probability of progressing into active TB disease, 5% during the first two years of acquiring the infection, and 5% during the rest of the individual's lifetime. The detection of LTBI and prevention before it becomes infectious is a crucial component of the WHO-End TB strategy. It has been reported from mathematical models that approximately 30% of the population worldwide are LTBI carriers [2]. Previous studies have documented the rates of LTBI to be 31.2% in Ethiopia [3], 49% in Uganda [4], 55.2% in South Africa [5], 11.2% in Spain [6], 50% in India [7], 51% in Korea [8], and 7.6% in England [9];however, very few studies have been undertaken to estimate the prevalence of LTBI in the Middle East and North Africa (MENA) region.

In previous studies, it has been observed that patients belonging to lower socioeconomic groups, refugees, and migrants [10], patients with abnormal immune responses (post-organ transplant, hemodialysis patients, people living with HIV, etc.), and chronic inflammatory conditions have an increased risk of acquiring TB and its progression to active disease [11–13];further, LTBI in people living with HIV has a 10% probability of progressing into active TB, when left untreated, annually; furthermore, it has been shown that a significant geographical variation in TB infection rates persists across the world, implying that health care workers (HCW) in various countries encounter different risks of acquiring TB [14]. In 2018, 87% of new TB cases occurred in the top thirty high TB burden countries, of which eight countries accounted for two-thirds of all new TB cases, they include India, China, Indonesia, Philippines, Pakistan, Nigeria, Bangladesh, and South Africa, while the occurrence was extremely low in the MENA regions [1], it has also been reported that HCW are at particular risk of LTBI, and hence, annual screening is performed in most standardized health care facilities. In addition, the prevalence of LTBI in HCW has been reported to be higher than that of other community groups around the world [15, 16].

Currently, the direct diagnosis of LTBI is not fully possible [17]. The diagnosis of memory T-cell response against LTBI is performed by either the tuberculin skin test (TST) or interferon-gamma release assays (IGRA) [18]. At present, no gold standard test has been developed to measure LTBI; however, there are increasing advancements in this field looking into tumor necrosis factor, chemokines, interleukin growth factors, and other factors that could enhance LTBI diagnosis [19]. With TST, TB-purified protein derivative (PPD) stimulates a type IV hypersensitivity-delayed type reaction [20–22], its advantage is that it is inexpensive and generally accepted especially in low economic countries including Africa [3], but has several disadvantages, as it has demonstrated poor response in individuals with reduced immunity and those with active TB, requires two-step verification, is operative dependent, and exhibits low specificity in determining reactivation of TB in Bacillus Calmette-Guérin (BCG) vaccinated individuals, it can also cause false-positive results in patients sensitized to naturally existing nontuberculous mycobacteria [18, 23].

On the other hand, IGRA has greater specificity compared to TST [17], it involves only one blood test after incubation with *Mycobacteria tuberculosis*-specific antigens, following which T-cell mediated immune response and interferon- (IFN-) gamma release are measured. The QuantiFERON®-TB-Gold-in-Tube (QFT-GIT) and T-SPOT.TB assay tests are the two commercially available IGRA, in which the former is based on ELISA (enzyme-linked immunosorbent assay) and comprises of peptides from the ESAT-6, CFP-10, and TB7.7 antigens of TB. T-SPOT.TB assay is preferred in immunocompromised patients [24–26]. IGRA provides more conclusive results that would help in decision-making, with only a single visit required for the test, it also eliminates false-positive results in people vaccinated with BCG or sensitized with nontuberculous mycobacteria.

Several previous studies have documented the prevalence of LTBI in many countries of the Middle East and North Africa, in a wide range of population, including HCW, household contacts, people living with HIV, prisoners, refugees, and in patients with varied health problems; however, to our knowledge, there are no published studies that have assessed the overall prevalence within the whole MENA region; hence, we performed a systematic review to evaluate the prevalence of LTBI in the MENA region in different population groups belonging to various age groups.

## 2. Methods

### 2.1. Criteria for Considering Studies

#### 2.1.1. Inclusion Criteria

Studies based on the incidence or prevalence of LTBI among people of all ages, origin, socioeconomic, and educational backgrounds, in countries located in the Middle East and North Africa, that are cross-sectional, observational, cohort, prospective, and retrospective studies, with LTBI detection performed with either TST or IGRA or both.

#### 2.1.2. Exclusion Criteria

Systematic reviews, case reports, case series, editorials, letters to the editors, and randomized controlled trials.

### 2.2. Search Strategy

The author searched PubMed and Google Scholar databases for articles published between January 1, 2000 and November 30, 2018, in the English language. The use of medical subject heading (MeSH) terms for LTBI was employed in the database search combined with the following search terms: (latent tuberculosis OR TB OR LTBI OR *Mycobacterium tuberculosis*) AND (Prevalence OR Epidemiology OR “Country name”). The Middle East countries included were Iran, Iraq, Saudi Arabia, Yemen, Syria, Jordan, United Arab Emirates, Israel, Lebanon, Oman, Kuwait, Qatar, Bahrain, Palestine, Cyprus, and Turkey. North African countries included were Egypt, Libya, Algeria, Morocco, Tunisia, Sudan, Western Sahara, and Mauritania. A broad search strategy was used to ensure that all relevant studies were identified, with no filters included in the searches. Following this, the author independently analyzed the title of the study and its abstract and keywords outlining the record, based on which studies were either included or excluded. No minimal sample size was required to be included in the analysis; however, a sample size of ≥200 was considered as adequate, and a sample size of <200 was considered as inadequate.

### 2.3. Data Extraction

#### 2.3.1. Study Selection and Data Extraction

A detailed search of PubMed and Google Scholar databases by employing various search terms was performed. The duplicate citations were removed, and the studies for inclusion in the review were selected. The initial screening was based on the citation titles and abstracts, following which, the articles were selected and picked up and their complete text obtained, reviewed, and assessed for their eligibility for inclusion. The bibliographic information of the included studies was also screened to identify additional relevant articles for inclusion; furthermore, the data from relevant studies were abstracted using a data extraction form, and the applicable items for the review were reported in the PRISMA (Preferred Reporting Items for Systematic Reviews and Meta-Analyses) checklist. The following key information has been presented in the data extraction template: first author, period of study and year of publication, country where the research was conducted, study design, number of participants, age at assessment, tools used for assessment, and key findings.

#### 2.3.2. Quality (Risk of Bias) Assessment

The Mirza and Jenkins [27] checklist were referred to for investigating the quality of included studies. The assessment was based on the following nine criteria: clear study aims, adequate sample size, representative sample, inclusion and exclusion criteria, adequate assessment of outcome, response rate reported, adequate description of data, appropriate statistical analysis, and appropriate informed consent obtained. A final total score was calculated for each of the criteria, scored 0 if absent and 1 if present. Thus, the minimum and maximum obtainable scores would be 0 and 9, respectively.

### 2.4. Statistical Analysis

Analysis was performed using STATA software. The effect sizes were reported as proportions with 95% confidence intervals. The heterogeneity of effects was assessed and quantified by the *I*^2^. The *I*^2^ values greater than 50% were considered to represent substantial heterogeneity. The random-effects model was subjected in cases exhibiting substantial heterogeneity. Subgroup analysis based on sex (male and female), by age strata, and by quality score of the studies (<5 and ≥5) was also performed. A *p* value less than 0.05 was considered statistically significant for all the analyses undertaken.

## 3. Results

### 3.1. Search Results and Study Selection

**Figure 1:**
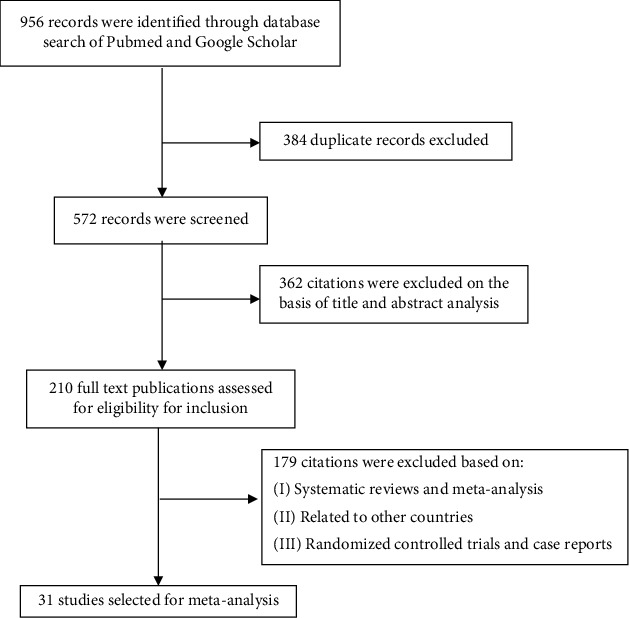
Flowchart for study selection.

The database search resulted in a total of 956 citations, of which 384 citations were eliminated due to their duplication, and the rest of the 572 citations were examined. After screening, examination of titles and abstracts resulted in the elimination of 362 citations from the study. Following this, 210 full-text citations were retrieved, and after subjecting them to inclusion and exclusion criteria, a total of 31 studies were identified (Figure 1).

### 3.2. Study Characteristics

Thirty-one studies representing 12,439 subjects from ten countries within the MENA region were included: thirteen from Turkey, five from both Iran and Saudi Arabia, two from Egypt, and one each from Syria, Israel, Oman, Qatar, Tunisia, and United Arab Emirates. These studies were conducted between 2005 till 2018. The sample size ranged from 34 to 2,650 (Table 1).

### 3.3. Publication Bias

From the 31 studies, the minimal checklist score was 5 in two studies, while the highest was 9. Details of all included studies clarity, adequacy of sample size, and other details are outlined in Table 2.

### 3.4. Prevalence of LTBI

The prevalence of LTBI was assessed in 31 studies using random-effects model. A total of 3,981 events were observed among the 12,439 subjects. The proportion of LTBI ranged from 0.44% to 88.15%. The overall prevalence was observed to be 41.78% (95% CI 31.18% to 52.78%, *I*^2^ = 99.31%).

The subgroup analyses revealed the existence of heterogeneity. In the gender-based subgroup analysis, some of the studies failed to mention the gender-based prevalence of LTBI, and hence 14 and 15 studies were excluded from the subgroup analysis of males and females, respectively; hence, the subgroup analysis of males was performed with 17 studies, and that of females with 16 studies. The analysis revealed that the proportion of LTBI ranged from 0.32% to 86.04% and from 0.54% to 90.90% in males and females, respectively. The overall prevalence was estimated to be 33.12% (95% CI 18.97% to 49.04%, *I*^2^ = 99.25%) and 32.65% (95% CI 19.79% to 47%, *I*^2^ = 98.89%) in males and females, respectively.

For the evaluation of age-based prevalence, the WHO classification for age groups was utilized, and the age range for children, adolescents, and adults was taken as <10 years, between 10 and 19 years, and >19 years, respectively; further, three studies, Shitrit et al. [28], Yilmaz et al. [29], and Jam et al. [30], were excluded from this subgroup analysis as the age of subjects in those studies overlapped the age range for children, adolescents, and adults, i.e., 12 years and above, 13 to 67 years, and 1 month to above 60 years, respectively. Moreover, there was no differentiation in the age range for the prevalence of LTBI in these studies; hence, the subgroup analysis of children, adolescents, and adults was performed with 1, 2, and 27 studies, respectively. The prevalence of LTBI in children was observed to be 0.44% (95% CI -0.05% to 0.9%); the prevalence of LTBI in adolescents and adults ranged from 2.46% to 3.55% and 6.93% to 88.15%, respectively. The overall prevalence was observed to be 3.37% (95% CI 2.23% to 4.74%, *I*^2^ = 0%) and 43.81% (95% CI 33.09% to 54.82%, *I*^2^ = 99.18%) for adolescents and adults, respectively.

## 4. Discussion

After screening 956 studies, a total of 31 scientific papers from ten countries within the MENA region were included in this systematic review [28–58]. The subjects included in these studies were healthcare workers, laboratory staff, medical school students, people living with HIV, and patients with chronic inflammatory diseases. The detection of LTBI in these studies was performed by TST or IGRA or both; furthermore, the studies covered the incidence of LTBI among populations belonging to varying age groups, including children, adolescents, and adults.

In the present study, LTBI prevalence was evaluated by employing the random effects model since high heterogeneity was encountered among studies. The existence of high heterogeneity may have possibly been due to variations in study settings, subjects or participants, methodologies involved, exposure to TB patients, and the control measures taken across the studies.

The overall prevalence of LTBI in the MENA region was found to be 41.78%. In the gender-based subgroup analyses, the prevalence of LTBI was found to be 33.12% and 32.65% in males and females, respectively. As for the age-based prevalence, it was assessed to be 0.44%, 3.37%, and 43.81% in children, adolescents, and adults, respectively; therefore, this systematic review implies a high prevalence of LTBI in the MENA region irrespective of gender, and in order to achieve the WHO End TB 2035 objective, there is an immediate need to scale up measures to stop TB disease and enhance LTBI detection within the MENA region.

There are some strengths and limitations within this study that needs to be highlighted; first, as per our findings, this is the first systematic review on the epidemiology and prevalence of LTBI in the MENA region. As for limitations, studies published in English alone have been included, therefore, other reports from countries with high TB incidence that are published in native or other languages other than English, in national or local journals, have not been included; additionally, studies published in journals indexed in PubMed and Google Scholar were included, while other studies may exist that were published in other indexing databases.

To conclude, this review indicates a high prevalence of LTBI in the MENA region despite the high heterogeneity observed. Future studies should aim towards more rigorous assessment of LTBI prevalence within the MENA region to reach exact estimates as the first important step to hamper TB disease diffusion in these countries.

## Figures and Tables

**Table 1:** Study characteristics.

Study/reference number	Duration of study	Year	Country	Study population	TST and/or QFT	Study design	Sample size	Age (mean ± SD)	Tools	LTBI	Prevalence (95% CI)	Outcome
Nasehi et al. [31]	October to December 2013	2016	Iran	TB laboratory staff and low-risk healthcare workers	TST	Cross-sectional	1006	38.06 ± 7.76 and 37.31 ± 7.32	ANOVA, logistic regression	791	78.62% (75.96, 81.12)	TB laboratory staff the OR of developing LTBI
Mamani et al. [32]	March 2013, 6 months	2016	Iran	Prisoners	TST	Cross-sectional	1208	18-60 years	Wilson procedure with continuity correction	756	62.58% (59.78, 65.32)	High prevalence of LTBI
Bukhary et al. [33]	December 2015	2018	Saudi Arabia	Healthcare workers working in hajj pilgrimage	TST QFT-GIT	Cross-sectional	520	22-62 years	Standardized questionnaire, chi-square test, Fisher exact test	56	10.76% (8.23, 13.75)	Low prevalence of LTBI
Balkhy et al. [34]	July 2010 to March 2013	2017	Saudi Arabia	Primary healthcare workers	TST QFT-GIT	Cross-sectional	1369	<15 to ≥65 years	Chi-square test, McNemar test	146	10.66% (9.07, 12.42)	Low prevalence of LTBI
El-Helaly et al. [35]	August 2009 to May 2011	2014	Saudi Arabia	Preemployment screening of tertiary healthcare workers	TST QFT-GIT	Cross-sectional	1372	18-60 years	Kappa coefficient, chi-square test	421	30.68% (28.25, 33.20)	Fair agreement between TST and QFT-G tests
Hassan and Diab et al. [36]	January to June 2012	2014	Saudi Arabia	Laboratory personnel at a university hospital	QFT-GIT	Cross-sectional	134	21-60 years (33 ± 9.2)	Standardized questionnaire, chi-square test, Fisher's exact test	26	19.4% (13.08, 27.12)	Assessed risk factors involved with LTBI
Abbas et al. [37]	January 2008 to December 2009	2010	Saudi Arabia	Healthcare workers in tertiary care hospital	TST	Cross-sectional	2650	10 to >50 years	ANOVA	291	10.98% (9.81,12.23)	Highest LTBI rates in physicians and nurses
Warrington et al. [38]	January 2016	2018	Syria	Syrian refugees entering Canada	QFT-GIT	Cross-sectional	99	5 to <50 years	Two-tailed independent *t*-tests	9	9.09% (4.24, 16.55)	Low prevalence of LTBI. No active TB
Mekaini et al. [39]	April to October 2013	2014	UAE	Pediatric population	QFT-GIT	Cross-sectional	669	1-19 years	Kruskal-Wallis one-way ANOVA, chi-square test, Fisher's exact test	3	0.44% (0.09, 1.30)	Low prevalence of LTBI
Shitrit et al. [28]	September and December 2002	2005	Israel	High school students and adults	TST	Cross-sectional	84	18.2 ± 11 years	Pearson correlation coefficient, Student's *t*-test	57	67.85% (56.77, 77.63)	High prevalence of LTBI
Khamis et al. [40]	January to June 2012	2016	Oman	Healthcare workers exposed to active TB in tertiary care hospital	TST QFT-GIT	Cross-sectional	291	20 to 65 years	Descriptive statistics	123	42.26% (36.52, 48.17)	High prevalence of LTBI among healthcare workers
Garcell et al. [41]	August 2012 to May 2013	2014	Qatar	Healthcare workers in community hospital	TST QFT-GIT	Cross-sectional	202	39 ± 6.5 years	Test of independence, Student's *t*-test, and Wilcoxon Mann–Whitney	14	6.93% (3.84, 11.35)	Low prevalence of LTBI
Gunluoglu et al. [42]	September to November 2011	2015	Turkey	Chronic renal failure patients undergoing regular hemodialysis	TST QFT-GIT	Cross-sectional	44 (TST); 50 (QFT-GIT)	62.2 years (mean age)	Kappa statistic, Mann–Whitney *U*-test, chi-square, Fisher's exact test, Wilks' lambda test	16 (TST+); 27 (QFT-GIT+)	50% (37.23, 62.76)	High prevalence of LTBI
Duman et al. [43]	Not available	2014	Turkey	Psoriasis patients	TST QFT-GIT	Cross-sectional	61 (psoriasis); 40 (psoriatic arthritis)	44.6 ± 13.1 years	Kolmogorov–Smirnov test, *t*-test, Mann–Whitney *U*-test, chi-square test, multivariable logistic regression, multiple linear regression	52 (psoriasis); 29 (psoriatic arthritis)	80.19% (71.08, 87.46)	High prevalence of LTBI
Babayigit et al. [44]	Not available	2014	Turkey	BCG vaccinated healthcare workers	TST QFT-GIT	Cross-sectional	64	21 to 51 years (32.01 ± 6.28)	Kolmogorov-Smirnov test, Shapiro Wilk test, Mann–Whitney *U*-test, Fisher exact test, Pearson chi-square test, logistic regression analysis	32	50% (37.23, 62.76)	High prevalence of LTBI
Yilmaz et al. [29]	Not available	2012	Turkey	Patients with systemic lupus erythematosus	TST QFT-GIT	Cross-sectional	78	13 to 67 years	Cohen's kappa analysis, chi-square test, Mann–Whitney *U*-test	41	52.56% (40.93, 63.99)	High prevalence of LTBI
Hanta et al. [45]	Not available	2012	Turkey	Patients with rheumatologic diseases	TST QFT-GIT	Cross-sectional	90	41.9 ± 11.9 years	Chi-square test or Fisher's exact test	66	73.33% (62.96, 82.10)	High prevalence of LTBI
Soysal et al. [46]	May 2006 to May 2007	2012	Turkey	Hemodialysis patients	TST T-SPOT.TB	Cross-sectional	411	19 to 84 years	Student's *t*-test, chi-square test or Fisher's exact test, logistic regression analysis	61	14.84% (11.54, 18.65)	Use of T-SPOT.TB in patients with negative TST for diagnosis of LTBI
Caglayan et al. [47]	August 2005	2011	Turkey	Healthcare workers of tertiary care hospital	TST QFT-GIT	Cross-sectional	78	30.51 ± 8.57 years	ANOVA	59	75.64% (64.60, 84.65)	High prevalence of LTBI
Karadag et al. [48]	Not available	2010	Turkey	Patients with Takayasu arteritis	TST QFT-GIT	Cross-sectional	94	40.2 ± 12.1 years	Student's *t*-test, Wilcoxon rank-sum test, chi-square test, Fisher's exact test	55	58.51% (47.88, 68.58)	High prevalence of LTBI
Inanc et al. [49]	March 2007 to June 2008	2009	Turkey	Patients with rheumatoid arthritis and Ankylosing spondylitis	TST QFT-GIT	Cross-sectional	140	55.4 ± 11.2 years	Chen's kappa analysis, Mann–Whitney *U*-test, chi-square test	85	60.71% (52.11, 68.85)	High prevalence of LTBI
Seyhan et al. [50]	Not available	2010	Turkey	Hemodialysis patients	TST QFT-GIT	Cross-sectional	100	56.2 ± 15.3 years	Student *t*-test, Mann–Whitney *U*-test, chi-square test	56	56% (45.71, 65.91)	High prevalence of LTBI
Hanta et al. [51]	April 2005 to January 2008	2008	Turkey	Patient with rheumatoid arthritis, ankylosing arthritis, and psoriatic arthritis	TST	Cross-sectional	192	43.1 ± 12.7 years	Fisher's exact test	129	67.18% (60.05, 73.77)	TST can be used for diagnosis of LTBI in rheumatologic disease before anti-TNF therapy.
Ozdemir et al. [52]	June to August 2005	2007	Turkey	Healthcare workers in Duzce University hospital	TST QFT-GIT	Cross-sectional	76	18 to 50 years (30.4 ± 5.4)	Cohen's kappa, chi-square test, Student's *t*-test	67	88.15% (78.70, 94.44)	High prevalence of LTBI
Bozkanat et al. [53]	March 2008	2016	Turkey	Healthcare workers in specialist tuberculosis hospital	TST QFT-GIT	Cross-sectional	34	33.0 ± 5.8 years	Kappa test	23	67.64% (49.47, 82.61)	High prevalence of LTBI
Hasanain et al. [54]	December 2015 to January 2017	2018	Egypt	Patients with erectile dysfunction	TST QFT-GIT	Cross-sectional	97	47.9 ± 13.6 years	Chi-square test, Fisher's exact test	29	29.89% (21.02, 40.04)	Prevalence of LTBI was high in patients with high-grade ED
El-Sokkary et al. [55]	August 2012 to January 2013	2015	Egypt	Healthcare providers	TST QFT-GIT	Cross-sectional	132	35.2 ± 8.99 years	Chi-square test, Fisher's exact test	78	59.09% (50.19, 67.56)	High prevalence of LTBI
Slouma et al. [56]	2007 to 2014	2017	Tunisia	Patients with chronic inflammatory diseases receiving biologic agents since at least 6 months	TST QFT-GIT	Cohort	113	47.67 ± 13.5 years	Student's *t*-test, ANOVA	23	20.35% (13.36, 28.95)	Low prevalence of LTBI
Khazraiyan et al. [57]	January to May 2013	2016	Iran	HIV positive patients	TST QFT-GIT	Cross-sectional	130	19 to 71 years (37.1 ± 8.6)	Chi-square test, Fisher's exact test	38	29.23% (21.58, 37.84)	Low prevalence of LTBI
Jam et al. [30]	January 2006 to February 2007	2010	Iran	Patients with HIV/AIDS	TST	Cross-sectional	262	1 month to >60 years	Chi-square test	63	24.04% (19, 29.68)	Medium prevalence of LTBI
Amiri et al. [58]	June to August 2012	2014	Iran	Homeless people of Tehran	QFT-GIT	Cross-sectional	593	Not available	Logistic regression and chi-square test	277	46.71% (42.63,50.81)	High prevalence of LTBI
											[Overall prevalence 41.78% (31.18, 52.78)]	

QFT-GIT: QuantiFERON-TB Gold In-Tube; TST: tuberculin skin test; LTBI: latent tuberculosis infection; OR: odds ratio; ANOVA: analysis of variance; HIV: human immunodeficiency virus; AIDS: acquired immunodeficiency syndrome; TNF: tumor necrosis factor.

**Table 2:** Quality assessment of the studies included in the review.

Study	Clear study aims	Adequate sample size	Representative sample	Inclusion and exclusion criteria	Adequate assessment of outcome	Response rate reported	Adequate description of data	Appropriate statistical analysis	Appropriate informed consent obtained	Total score
Nasehi et al., 2016 [31]	1	1	1	1	1	1	1	1	1	9
Mamani et al., 2016 [32]	1	1	1	1	1	1	1	1	1	9
Bukhary et al., 2018 [33]	1	1	1	1	1	0	1	1	1	8
Balkhy et al., 2017 [34]	1	1	1	1	1	0	1	1	1	8
El-Helaly et al., 2014 [35]	1	1	1	1	1	0	1	1	0	7
Hassan and Diab, 2014 [36]	1	0	1	1	0	0	1	1	1	6
Abbas et al., 2010 [37]	1	1	1	1	1	0	1	1	0	7
Warrington et al., 2018 [38]	1	0	1	1	0	0	1	1	0	5
Mekaini et al., 2014 [39]	1	1	1	1	1	0	1	1	1	8
Shitrit et al., 2005 [28]	1	0	1	1	0	1	1	1	1	7
Khamis et al., 2016 [40]	1	1	1	1	1	0	1	1	0	7
Garcell et al., 2014 [41]	1	1	1	1	1	0	1	1	0	7
Gunluoglu et al., 2015 [42]	1	0	1	1	0	1	1	1	1	7
Duman et al., 2014 [43]	1	0	1	1	0	1	1	1	1	7
Babayigit et al., 2014 [44]	1	0	1	1	0	1	1	1	1	7
Yilmaz et al., 2012 [29]	1	0	1	1	0	1	1	1	1	7
Hanta et al., 2012 [45]	1	0	1	1	0	1	1	1	0	6
Soysal et al., 2012 [46]	1	0	1	1	0	0	1	1	0	5
Caglayan et al., 2011 [47]	1	0	1	1	0	1	1	1	0	6
Karadag et al., 2010 [48]	1	0	1	1	0	1	1	1	0	6
Inanc et al., 2009 [49]	1	1	1	1	1	1	1	1	1	9
Seyhan et al., 2010 [50]	1	0	1	1	0	1	1	1	1	7
Hanta et al., 2008 [51]	1	0	1	1	0	1	1	1	0	6
Ozdemir et al., 2007 [52]	1	0	1	1	0	1	1	1	1	7
Bozkanat et al., 2016 [53]	1	0	1	1	0	1	1	1	0	6
Hasanain et al., 2018 [54]	1	0	1	1	0	0	1	1	1	6
El-Sokkary et al., 2015 [55]	1	0	1	1	0	1	1	1	1	7
Slouma et al., 2017 [56]	1	0	1	1	0	0	1	1	0	5
Khazraiyan et al., 2016 [57]	1	0	1	1	0	0	1	1	1	6
Jam et al., 2010 [30]	1	1	1	1	1	0	1	1	1	8
Amiri et al., 2014 [58]	1	1	1	1	1	0	1	1	0	7

^∗^A sample size of ≥200 was considered as adequate and a sample size of <200 was considered as inadequate. ^†^A response rate of <50% was considered as low = 0, and>50% was considered as high = 1.

## Data Availability

All data are included in the manuscript.
